# Coordinated action of human papillomavirus type 16 E6 and E7 oncoproteins on competitive endogenous RNA (ceRNA) network members in primary human keratinocytes

**DOI:** 10.1186/s12885-021-08361-y

**Published:** 2021-06-07

**Authors:** Brigitta László, László Antal, Eszter Gyöngyösi, Anita Szalmás, Szilárd Póliska, György Veress, József Kónya

**Affiliations:** 1 Department of Medical Microbiology, Faculty of Medicine, University of Debrecen, Nagyerdei krt. 98, Debrecen, H-4032 Hungary; 2 Department of Hydrobiology, University of Debrecen, Egyetem tér 1, Debrecen, H-4032 Hungary; 3 Genomic Medicine and Bioinformatics Core Facility, Department of Biochemistry and Molecular Biology, Faculty of Medicine, University of Debrecen, Nagyerdei krt. 98, Debrecen, H-4032 Hungary

**Keywords:** HPV16, E6, E7, microRNA, Long non-coding RNA, Competing endogenous RNA (ceRNA)

## Abstract

**Background:**

miRNAs and lncRNAs can regulate cellular biological processes both under physiological and pathological conditions including tumour initiation and progression. Interactions between differentially expressed diverse RNA species, as a part of a complex intracellular regulatory network (ceRNA network), may contribute also to the pathogenesis of HPV-associated cancer. The purpose of this study was to investigate the global expression changes of miRNAs, lncRNAs and mRNAs driven by the E6 and E7 oncoproteins of HPV16, and construct a corresponding ceRNA regulatory network of coding and non-coding genes to suggest a regulatory network associated with high-risk HPV16 infections. Furthermore, additional GO and KEGG analyses were performed to understand the consequences of mRNA expression alterations on biological processes.

**Methods:**

Small and large RNA deep sequencing were performed to detect expression changes of miRNAs, lncRNAs and mRNAs in primary human keratinocytes expressing HPV16 E6, E7 or both oncoproteins. The relationships between lncRNAs, miRNAs and mRNAs were predicted by using StarBase v2.0, DianaTools-LncBase v.2 and miRTarBase. The lncRNA-miRNA-mRNA regulatory network was visualized with Cytoscape v3.4.0. GO and KEEG pathway enrichment analysis was performed using DAVID v6.8.

**Results:**

We revealed that 85 miRNAs in 21 genomic clusters and 41 lncRNAs were abnormally expressed in HPV E6/E7 expressing cells compared with controls. We constructed a ceRNA network with members of 15 lncRNAs – 43 miRNAs – 358 mRNAs with significantly altered expressions. GO and KEGG functional enrichment analyses identified numerous cancer related genes, furthermore we recognized common miRNAs as key regulatory elements in biological pathways associated with tumorigenesis driven by HPV16.

**Conclusions:**

The multiple molecular changes driven by E6 and E7 oncoproteins resulting in the malignant transformation of HPV16 host cells occur, at least in part, due to the abnormal alteration in expression and function of non-coding RNA molecules through their intracellular competing network.

**Supplementary Information:**

The online version contains supplementary material available at 10.1186/s12885-021-08361-y.

## Background

Human papillomaviruses (HPVs) are small, double-stranded circular DNA viruses with significant oncogenic potential. HPVs infect epithelial cells of skin and mucous membranes mainly in the anogenital and head and neck region. High-risk HPV-types, such as HPV16, 18, 31, 33, 45, 51, 58 etc., are main etiologic factors of numerous different carcinomas including cervical, vulvar, penile, anal and head and neck cancers. Nearly 100% of cervical carcinomas are positive for high-risk HPVs, HPV16 being the most prevalent type detected in these malignancies [1, 2]. E6 and E7 early viral proteins are the major oncoproteins of high-risk HPVs. The transforming activity of E6 and E7 is mainly mediated through binding and inactivation of cellular tumour suppressor proteins p53 and pRb, respectively, although they are able to directly or indiretly interact with numerous cellular factors involved in regulation of cell cycle, cell death or cellular differentiation, thereby promote malignant transformation [3–5].

High-throughput RNA-seq studies have revealed the great complexity of human transcriptome by demonstrating that only approximately 2% of the human genome encodes protein-coding sequences. A significant portion of the human genes is actively transcribed but not translated into functional proteins suggesting their crucial regulatory role in biological processes under different conditions. Among these transcripts, several classes of non-coding RNA molecules have been identified including microRNAs (miRNAs), small nucleolar RNAs (snoRNAs), PIWI-interacting RNAs (piRNAs) and long non-coding RNAs (lncRNAs) [6, 7]. The approximately 18–25 nt miRNAs are the most intensively studied and best-known subtype of non-coding RNAs. miRNAs downregulate the expression of their targets post-transcriptionally by binding to the 3′-UTR of mRNAs leading to the translational inhibition or degradation of their target. Expression of miRNAs may vary in different tissues, developmental stages or pathological conditions [8–12]. lncRNAs are a diverse group of non-coding transcripts longer than 200 nt which are thought to have important function in gene expression regulation. Their ability to interact with DNA, RNA or proteins suggests their multiple functions in diverse biological processes. They have been linked to epigenetic mechanisms, splicing, translational regulation and protein stability, although the accurate mechanism of their action remains largely unknown [9, 13–16].

Emerging evidence shows the aberrant function of non-coding RNAs especially miRNAs and lncRNAs in various diseases and tumours including HPV-induced cervical cancers. The non-coding transcripts involved in cell cycle, differentiation, proliferation, migration and apoptosis tend to have altered expression during the initiation and progression of tumorigenesis [9, 15, 17–27]. Although several details of the complex pathogenesis of HPV-induced carcinogenesis have been explored primarily by focusing on oncogenic activity of E6 and E7 proteins of high-risk HPV types, the complete mechanism is not fully understood [3, 28]. An increasing number of studies have reported both abnormal upregulation and downregulation of numerous miRNAs in cervical cancer cell lines and tissue samples, strongly suggesting the oncogenic or tumour suppressor function of certain miRNAs in HPV-associated carcinogenesis [29–38]. As for the lncRNAs, despite the growing body of evidence regarding their importance in oncogenesis, only a limited number of studies have directly linked certain lncRNAs to HPV-associated cervical carcinoma [15, 21, 39–43]. Thus, the modulation of a wide range of host lncRNAs by E6 and E7 oncoproteins of high-risk HPVs remains to be extensively investigated.

According to the newly established competing endogenous RNA (ceRNA) hypothesis, all types of RNAs, both protein coding and non-coding transcripts are able to “talk to each other” and influence the functions of the others as a part of a complex intracellular regulatory network. This communication is thought to be mediated by miRNAs through binding to microRNA response elements (MREs) on various RNA transcripts [44]. Recent studies have proved that lncRNAs have the ability to regulate miRNA activity by acting as decoys for them, leading to the alteration of protein turnover. In this context, the altered interaction between lncRNAs and miRNAs by perturbation in their levels may contribute to the pathogenesis of cancer [9, 45–49].

In the current study, we performed high-throughput new generation RNA sequencing to detect cellular lncRNA and miRNA expression changes in primary human keratinocytes expressing HPV16 E6, E7 and E6/E7 oncoproteins. In addition to non-coding RNAs, mRNA expression changes were measured and we constructed a ceRNA network of coding and non-coding genes to suggest a regulatory network associated with HPV16 infection. We also refined the mRNAs of this ceRNA network by key biological pathways involved in cancer development as determined by Gene Ontology (GO) and Kyoto Encyclopedia of Genes and Genomes (KEGG) analysis.

## Methods

### Cell culture and RNA isolation

Primary human foreskin keratinocytes (HFK) transduced with LXSN-based retroviral vectors encoding HPV16 oncogenes were obtained from a previous study of our research group [50]. HPV16 E6, HPV16 E7 and HPV16 E6/E7 expressing cells and LXSN control cells were cultured in Defined Keratinocyte-Serum Free Medium (DK-SFM, Invitrogen) as described previously [51]. Two independent, passage-matched HFK populations were used in our experiments. Total RNA was isolated from each transduced keratinocyte cultures using TriReagent (Sigma-Aldrich) according to the manufacturer’s instructions. HPV16 E6 and E7 specific reverse transcription PCR was performed to confirm the expression of viral oncogenes in all HFK samples [52].

### RNA sequencing and data analysis

RNA integrity was checked on an Agilent BioAnalyzer 2100 instrument using RNA 6000 Nano kit (Agilent Technologies) according to manufacturer’s protocol and samples with RNA integrity number > 7.0 were accepted to good quality and were used in the further experiments. A NanoDrop ND-1000 was used to determine RNA concentration. Global transcriptome analysis was performed on Illumina sequencing platform. Sequencing libraries for small RNA-Seq were generated from 1 μg total RNA using TruSeq Small RNA Sample Preparation Kit (Illumina, San Diego, CA, USA) according to the manufacturer’s protocol. Fragment size distribution and molarity of libraries were checked on Agilent BioAnalyzer DNA1000 chip (Agilent Technologies, Santa Clara, CA, USA). Then single read 50 bp sequencing run was performed on Illumina HiScan SQ instrument (Illumina, San Diego, CA, USA). cDNA library for RNA-Seq was generated from 1 μg total RNA using TruSeq RNA Sample Preparation Kit (Illumina, San Diego, CA, USA) according to the manufacturer’s protocol. Briefly, poly-A tailed RNAs were purified by oligodT conjugated magnetic beads and fragmented on 94 C degree for 8 min, then 1st strand cDNA was transcribed using random primers and SuperScript II reverse transcriptase (Lifetechnologies, Carslbad, CA, USA). Following this step second strand cDNA synthesized, double stranded cDNA end repaired and 3′ ends adenylated then Illumina index adapters were ligated. After adapter ligation enrichment PCR was performed to amplify adapter ligated cDNA fragments. Fragment size distribution and molarity of libraries were checked on Agilent BioAnalyzer DNA1000 chip (Agilent Technologies, Santa Clara, CA, USA). Single read 50 bp sequencing run was performed on Illumina HiScan SQ instrument (Illumina, San Diego, CA, USA). Quality control and alignment of raw reads to the reference human genome hg19 was done using FASTQC package. Quantification and Fold Change analysis (FC) were performed using StrandNGS software. Expressed genes were identified by filtering out the lowest 20 percentile of genes based on raw signal intensity. RNA sequencing and data analysis were carried out by Genomic Medicine and Bioinformatic Core Facility, Deparment of Biochemistry and Molecular Biology, Faculty of Medicine, University of Debrecen (Debrecen, Hungary).

### Quantitative real-time RT-PCR

For miRNA RT-qPCR, cDNA was synthesised using TaqMan MicroRNA Reverse Transcription Kit (Applied Biosystems) with miRNA-specific stem-loop primers. Mature miRNAs were detected on 7500 Real Time PCR System (Applied Biosystems) using TaqMan Universal Master Mix and MicroRNA Assays (Applied Biosystems) according to manufacturer’s protocol. PCR reactions were performed in triplicate, small RNA RNU44 was used as an endogenous control.

For large RNA RT-qPCR, the High-Capacity cDNA Reverse Transcription Kit (Applied Biosystems) was used to prepare cDNA. qPCR was performed using TaqMan Gene Expression Master Mix and Gene Expression Assays (Applied Biosystems) according to the manufacturer’s instructions. Each reaction was performed in triplicate and GAPDH (glyceraldehyde 3-phosphate dehydrogenase) was used as endogenous control. In data analysis, comparative C_t_ method was used to obtain the Relative Quantification (RQ) values with standard deviation and confidence intervals (7500 System SDS Software, version 2.0.6).

### Construction of ceRNA network, GO and KEGG pathway analysis

The construction of a regulatory network was a multistep process. At first, we identified miRNAs, lncRNAs and mRNAs which were differentially expressed in HFK-E6/E7 cells. ≥10 reads in raw data and at least ±1.5 fold change were applied as treshold cutoffs in data analysis. For each transcript, z-score and *p* value were calculated with Benjamini-Hochberg FDR correction of 0.10 to include the transcript into downstream analyses. lncRNA-miRNA interactions were identified using StarBase v2.0 and DianaTools-LncBase v.2 databases which provides experimentally supported interaction data [53, 54]. In the following step, mRNAs targeted by miRNAs were explored using the advanced search function of miRTarBase [55]. To establish the lncRNA-miRNA-mRNA network, we integrated lncRNA-miRNA and miRNA-mRNA interactions and the regulatory network was visualized with Cytoscape v3.4.0 [56]. In RNA-RNA interaction search, we used only the experimentally validated search module with default settings so only miRNA-lncRNA and miRNA-mRNA pairs were included in further analyses, which were previously experimentally validated and were differentially expressed in our experimental system, too. The lists of StarBase v2.0, DianaTools-LncBase v.2 and miRTarBase search results are shown in Additional file 1.

The coding genes involved in ceRNA network were input into the DAVID v6.8 (Database for Annotation, Visualization and Integrated Discovery) for GO (Gene Ontology) and KEEG (Kyoto Encyclopedia of Genes and Genomes) pathway enrichment analysis [57].

## Results

### Altered miRNA profile in HPV16 E6/E7 expressing keratinocytes

Next generation microRNA sequencing was used to investigate the alteration of miRNA expression levels in response to high risk HPV16 E6/E7 in undifferentiated primary human keratinocytes. Two independent passage-matched populations of human foreskin kerationcytes expressing HPV16 E6 and/or E7 or control vector (HFK-E6, HFK-E7, HFK-E6/E7 and HFK-LXSN) were used in our experiments. Cells were cultured in low-calcium, serum-free medium and harvested for RNA extraction at maximum 80% confluency after up to 5 passages to keep them undifferentiated and to avoid acquiring a transformed phenotype. In addition, E6 and E7 specific RT-PCR in this study (Additional file 2) and p53 and Rb Western blot analyses in our previous study verified the presence of functionally active oncoproteins in the transduced cells [58]. NGS analysis of cellular mRNAs - that was performed in this study - provides further evidence for the presence of functionally active E6 and E7 oncoproteins in the transduced cells. The effects of the HPV16 E6 and E7 on the level of well-known transcriptional targets of high-risk HPV oncoproteins (TERT, CDKN1A, CDKN2A, MDM2, etc.) are shown in Additional file 3. As a consequence, expression changes in RNA levels most probably represent the effect of HPV oncogenes rather than the effect of differentiation or transformation. Only miRNAs which matched the treshold criteria and showed consistent expression changes in the two independent experiments were involved in downstream analyses.

**Fig. 1:**
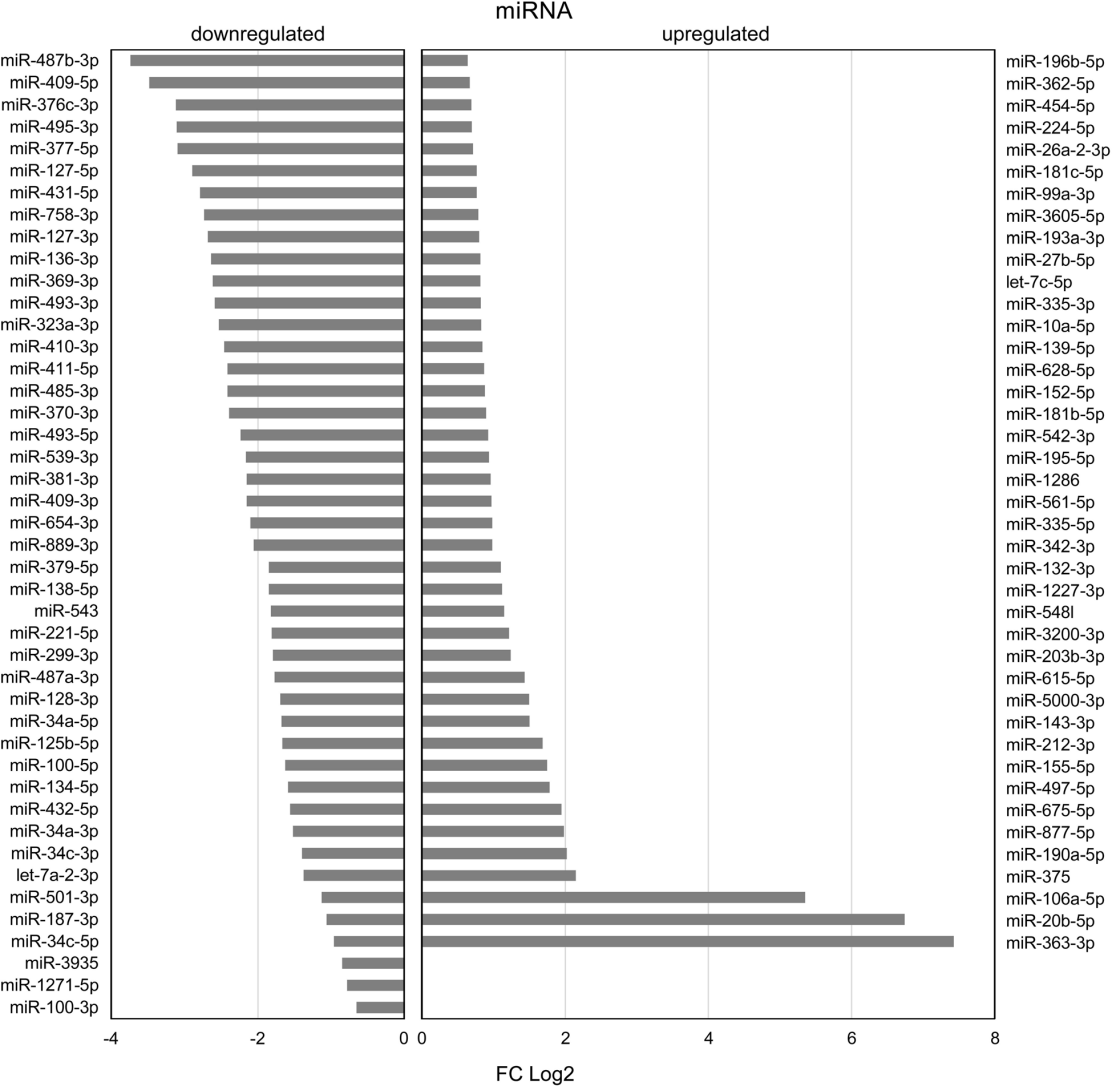
Differentially expressed miRNAs in HPV16 E6/E7 expressing keratinocytes compared to control vector cells. Average Log2 transformed FC values of up - and downregulated miRNAs are shown

85 differentially expressed miRNAs were detected in E6/E7 expressing HFKs compared to control vector cells (E6/E7 vs. LXSN). Of these dysregulated miRNAs, 44 miRNAs were downregulated (e.g. miR-34a-5p, miR-127-3p, miR-138-5p, miR-370-3p, miR-377-5p, miR-487b-3p, miR-654-3p) and 41 miRNAs (e.g. let-7c-5p, miR-10-5p, miR − 20b-5p, miR − 132-3p, miR-195-5p, miR-675-5p, miR-3605-5p) were upregulated (Fig. 1).

**Fig. 2:**
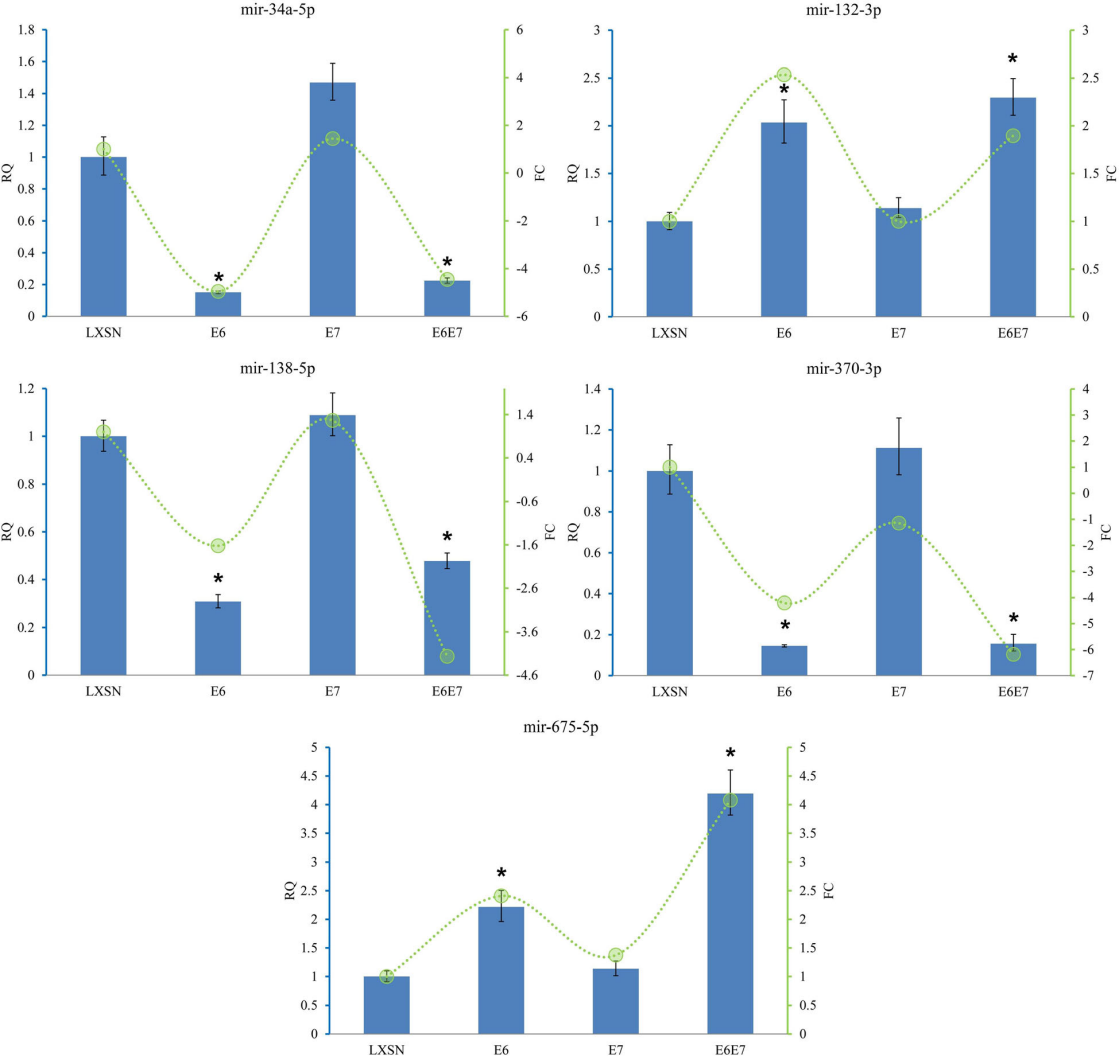
Validation of selected miRNAs identified by miRNA sequencing. The integrated results of sequencing and TaqMan analyses of miRNAs are shown. Green lines represent FC values obtained in miRNA sequencing. Altered expression of selected miRNAs was confirmed using TaqMan real-time assays in HPV16 E6, HPV16 E7, HPV16 E6/E7 and LXSN control cells in three independent experiments. In data analysis, comparative C_t_ method was used to obtain the Relative Quantification (RQ) values with standard deviation and confidence intervals (95%). The RQ value of LXSN control was set to 1. Representative graphs are shown. *p < 0.01

To validate the miRNA-seq results, altered expression of several miRNAs was confirmed using TaqMan real-time assays in three independent experiments. The RNA preparations used as template in 2 of 3 TaqMan assays were also used in the RNA sequencing experiments to verify the validity of RNA sequencing results. RNA preparation from an additional HFK population was used in the third TaqMan assay to further strengthen our observations in RNA sequencing. The downregulation of miR-34a-5p, miR-138-5p and miR-370-3p and the upregulation of miR-132-3p and miR-675-5p matched with the expression tendency seen in the sequencing results and their expression changes were statistically significant. The integrated results of sequencing and TaqMan analyses of selected miRNAs are shown in Fig. 2.

We investigated the changes in miRNA expression levels in HFK cells expressing only the E6 or E7 alone to understand the importance of individual oncoproteins in miRNA profile alteration. We considered the miRNA expression changes as a result of E6 oncoprotein if the expression changes were predominant in E6 vs LXSN and E6/E7 vs E7 comparisons in addition to E6/E7 vs LXSN results in both experiments. Similarly, E7 effect was supposed if the altered miRNA expression was observed mainly in E7 vs LXSN and E6/E7 vs E6 comparisons. In 56 of 85 dysregulated miRNAs, the modulation of expression was considered primarily the individual effect of HPV16 E6 oncoprotein. In contrast, miRNA expression alterations were observed to be driven predominantly by E7 only in 5 cases while up- or downregulation of 24 miRNAs was driven both by E6 and E7 together. Hence, majority of miRNA expression alterations in E6/E7 expressing keratinocytes occured as a result of the independent expression of HPV16 E6 or the cumulative effect of both E6 and E7 (Additional file 4).

### Effect of HPV16 E6/E7 and human miRNA clusters

We analyzed the effect HPV16 E6 and E7 oncoproteins by human miRNA clusters. The 85 miRNAs with altered expression were located to known miRNA clusters on the basis of miRBase, and 55 were identified as belonging to genomic clusters [59]. Out of the 55 clustered miRNAs, 20 were upregulated and 35 were downregulated. In total, the expression of 21 miRNA clusters were modulated by HPV16 E6/E7 oncoproteins. 21 differentially expressed miRNAs were members of small clusters containing only 2–4 miRNAs, while 34 miRNAs were members of larger clusters containing 6 to 22 miRNAs. According to the genomic location of miRNA clusters, chromosome X and chromosome 14 seem to have an importance consisting 5–5 clusters with altogether 38 altered miRNA members. The directions of miRNA expressional changes were either consistently up or consistently down within all but one clusters containing multiple miRNAs with altered expression (Table 1).

**Table 1:** miRNA clusters modulated in HPV16 E6/E7 expressing HFKs

miRNA clusters	chromosome location	FC_A	FC_B
hsa-mir-532	chrX: 50003148-50003238 [+]		
hsa-mir-188	chrX: 50003503-50003588 [+]		
hsa-mir-500a	chrX: 50008431-50008514 [+]		
**hsa-mir-362-5p**	chrX: 50008964-50009028 [+]	1.5723507	1.6101348
**hsa-mir-501-3p**	chrX: 50009722-50009805 [+]	-2.629669	-1.8152058
hsa-mir-500b	chrX: 50010672-50010750 [+]		
hsa-mir-660	chrX: 50013241-50013337 [+]		
hsa-mir-502	chrX: 50014598-50014683 [+]		
**hsa-miR-106a-5p**	chrX: 134170198-134170278 [-]	101.40113	16.514359
hsa-mir-18b	chrX: 134170041-134170111 [-]		
**hsa-mir-20b-5p**	chrX: 134169809-134169877 [-]	58.216805	197.02121
hsa-mir-19b-2	chrX: 134169671-134169766 [-]		
hsa-mir-92a-2	chrX: 134169538-134169612 [-]		
**hsa-mir-363-3p**	chrX: 134169378-134169452 [-]	175.14743	169.4563
hsa-mir-424	chrX: 134546614-134546711 [-]		
hsa-mir-503	chrX: 134546328-134546398 [-]		
**hsa-mir-542-3p**	chrX: 134541341-134541437 [-]	1.871955	1.919804
hsa-mir-450a-2	chrX: 134540508-134540607 [-]		
hsa-mir-450a-1	chrX: 134540341-134540431 [-]		
hsa-mir-450b	chrX: 134540185-134540262 [-]		
hsa-mir-452	chrX: 151959628-151959712 [-]		
**hsa-mir-224-5p**	chrX: 151958578-151958658 [-]	1.6341554	1.6087124
hsa-mir-222	chrX: 45747015-45747124 [-]		
**hsa-mir-221-5p**	chrX: 45746157-45746266 [-]	-4.637838	-2.646788
hsa-mir-181a-1	chr1: 198859044-198859153 [-]		
**hsa-mir-181b-1-5p**	chr1: 198858873-198858982 [-]	2.156177	1.6052669
**hsa-mir-143-3p**	chr5: 149428918-149429023 [+]	5.004603	1.6027163
hsa-mir-145	chr5: 149430646-149430733 [+]		
hsa-mir-23b	chr9: 95085208-95085304 [+]		
**hsa-mir-27b-5p**	chr9: 95085445-95085541 [+]	1.7026099	1.8156775
hsa-mir-3074	chr9: 95086014-95086094 [-]		
hsa-mir-24-1	chr9: 95086021-95086088 [+]		
hsa-mir-34b	chr11: 111512938-111513021 [+]		
**hsa-mir-34c-3p**	chr11: 111513439-111513515 [+]	-2.5223322	-2.7287412
**hsa-mir-34c-5p**	chr11: 111513439-111513515 [+]	-2.1740744	-1.7409978
**hsa-mir-100-3p**	chr11: 122152229-122152308 [-]	-1.3579255	-1.8180759
**hsa-mir-100-5p**	chr11: 122152229-122152308 [-]	-4.53056	-2.0923948
hsa-mir-10526	chr11: 122152103-122152156 [-]		
**hsa-let-7a-2-3p**	chr11: 122146522-122146593 [-]	-2.6796398	-2.4939332
hsa-mir-151b	chr14: 100109419-100109514 [-]		
**hsa-mir-342-3p**	chr14: 100109655-100109753 [+]	1.6633373	2.3525498
**hsa-mir-493-3p**	chr14: 100869060-100869148 [+]	-5.9027004	-6.0705276
**hsa-mir-493-5p**	chr14: 100869060-100869148 [+]	-6.4929733	-3.3994486
hsa-mir-337	chr14: 100874493-100874585 [+]		
hsa-mir-665	chr14: 100875033-100875104 [+]		
**hsa-mir-431-5p**	chr14: 100881007-100881120 [+]	-5.0243244	-9.432702
hsa-mir-433	chr14: 100881886-100881978 [+]		
**hsa-mir-127-3p**	chr14: 100882979-100883075 [+]	-6.343858	-6.488734
**hsa-mir-127-5p**	chr14: 100882979-100883075 [+]	-6.16204	-8.904066
**hsa-mir-432-5p**	chr14: 100884483-100884576 [+]	-2.1643238	-3.9932208
**hsa-mir-136-3p**	chr14: 100884702-100884783 [+]	-8.397293	-4.60158
**hsa-mir-379-5p**	chr14: 101022066-101022132 [+]	-2.061261	-6.2917047
**hsa-mir-411-5p**	chr14: 101023325-101023420 [+]	-4.652149	-6.0898
**hsa-mir-299-3p**	chr14: 101023794-101023856 [+]	-3.091891	-3.8669388
hsa-mir-380	chr14: 101025017-101025077 [+]		
hsa-mir-1197	chr14: 101025564-101025651 [+]		
**hsa-mir-323a-3p**	chr14: 101025732-101025817 [+]	-9.275676	-3.581611
**hsa-mir-758-3p**	chr14: 101026020-101026107 [+]	-9.79099	-4.4747305
hsa-mir-329-1	chr14: 101026785-101026864 [+]		
hsa-mir-329-2	chr14: 101027100-101027183 [+]		
hsa-mir-494	chr14: 101029634-101029714 [+]		
hsa-mir-1193	chr14: 101030052-101030129 [+]		
**hsa-mir-543**	chr14: 101031987-101032064 [+]	-3.4010804	-3.6604512
**hsa-mir-495-3p**	chr14: 101033755-101033836 [+]	-16.232431	-4.5614853
**hsa-mir-376c-3p**	chr14: 101039690-101039755 [+]	-20.097301	-3.7425764
hsa-mir-376a-2	chr14: 101040069-101040148 [+]		
**hsa-mir-654-3p**	chr14: 101040219-101040299 [+]	-4.637838	-3.9420242
**hsa-mir-381-3p**	chr14: 101045920-101045994 [+]	-3.9936907	-4.930125
**hsa-mir-487b-3p**	chr14: 101046455-101046538 [+]	-42.75289	-4.12974
**hsa-mir-539-3p**	chr14: 101047321-101047398 [+]	-8.502702	-2.346443
**hsa-mir-889-3p**	chr14: 101047901-101047979 [+]	-4.240309	-4.071979
hsa-mir-544a	chr14: 101048658-101048748 [+]		
hsa-mir-655	chr14: 101049550-101049646 [+]		
**hsa-mir-487a-3p**	chr14: 101052446-101052525 [+]	-4.637838	-2.5100238
hsa-mir-382	chr14: 101054306-101054381 [+]		
**hsa-mir-134-5p**	chr14: 101054687-101054759 [+]	-3.1471035	-2.8613925
hsa-mir-668	chr14: 101055258-101055323 [+]		
**hsa-mir-485-3p**	chr14: 101055419-101055491 [+]	-5.742084	-4.9056315
hsa-mir-323b	chr14: 101056219-101056300 [+]		
hsa-mir-154	chr14: 101059755-101059838 [+]		
hsa-mir-496	chr14: 101060573-101060674 [+]		
**hsa-mir-377-5p**	chr14: 101062050-101062118 [+]	-17.005402	-4.288576
hsa-mir-541	chr14: 101064495-101064578 [+]		
**hsa-mir-409-3p**	chr14: 101065300-101065378 [+]	-5.0722356	-3.8650641
**hsa-mir-409-5p**	chr14: 101065300-101065378 [+]	-15.459457	-8.040478
hsa-mir-412	chr14: 101065447-101065537 [+]		
**hsa-mir-369-3p**	chr14: 101065598-101065667 [+]	-10.821619	-3.4579167
**hsa-mir-410-3p**	chr14: 101065912-101065991 [+]	-6.966924	-4.316698
hsa-mir-656	chr14: 101066724-101066801 [+]		
**hsa-mir-203b-3p**	chr14: 104117418-104117503 [-]	2.5874128	2.1562526
hsa-mir-203a	chr14: 104117405-104117514 [+]		
**hsa-mir-212-3p**	chr17: 2050271-2050380 [-]	4.5279727	2.2936604
**hsa-mir-132-3p**	chr17: 2049908-2050008 [-]	2.4241295	1.8954328
**hsa-mir-152-5p**	chr17: 48037161-48037247 [-]	1.7206296	1.9654182
hsa-mir-10226	chr17: 48032882-48032951 [+]		
**hsa-mir-497-5p**	chr17: 7017911-7018022 [-]	6.468533	1.8376815
**hsa-mir-195-5p**	chr17: 7017615-7017701 [-]	2.2345843	1.6348059
**hsa-mir-181c-5p**	chr19: 13874699-13874808 [+]	1.8207724	1.5850542
hsa-mir-181d	chr19: 13874875-13875011 [+]		
hsa-mir-6789	chr19: 2235829-2235926 [-]		
**hsa-mir-1227-3p**	chr19: 2234062-2234149 [-]	2.1561775	2.1855226
**hsa-mir-99a-3p**	chr21: 16539089-16539169 [+]	1.6420119	1.7610615
**hsa-let-7c-5p**	chr21: 16539828-16539911 [+]	1.6335889	1.8939737

miRNAs in bold were found to be modulated by HPV16 E6 and E7

A and B represent the two independent sequencing experiments

### Long non-coding RNAs modulated by HPV oncoproteins

In addition to miRNA analysis, polyA-tailed long RNA (≥ 200 nt) sequencing was performed from the same HFK populations in two independent experiments to investigate the modulatory effect of HPV16 oncoproteins on long non-coding RNA expressions. The same HPV oncoprotein expressing keratinocytes, as well as the same validation criteria were applied in the analyses of our data. Only lncRNAs which matched the treshold criteria and showed consistent expression results in the two independent experiments were involved in downstream analyses.

**Fig. 3:**
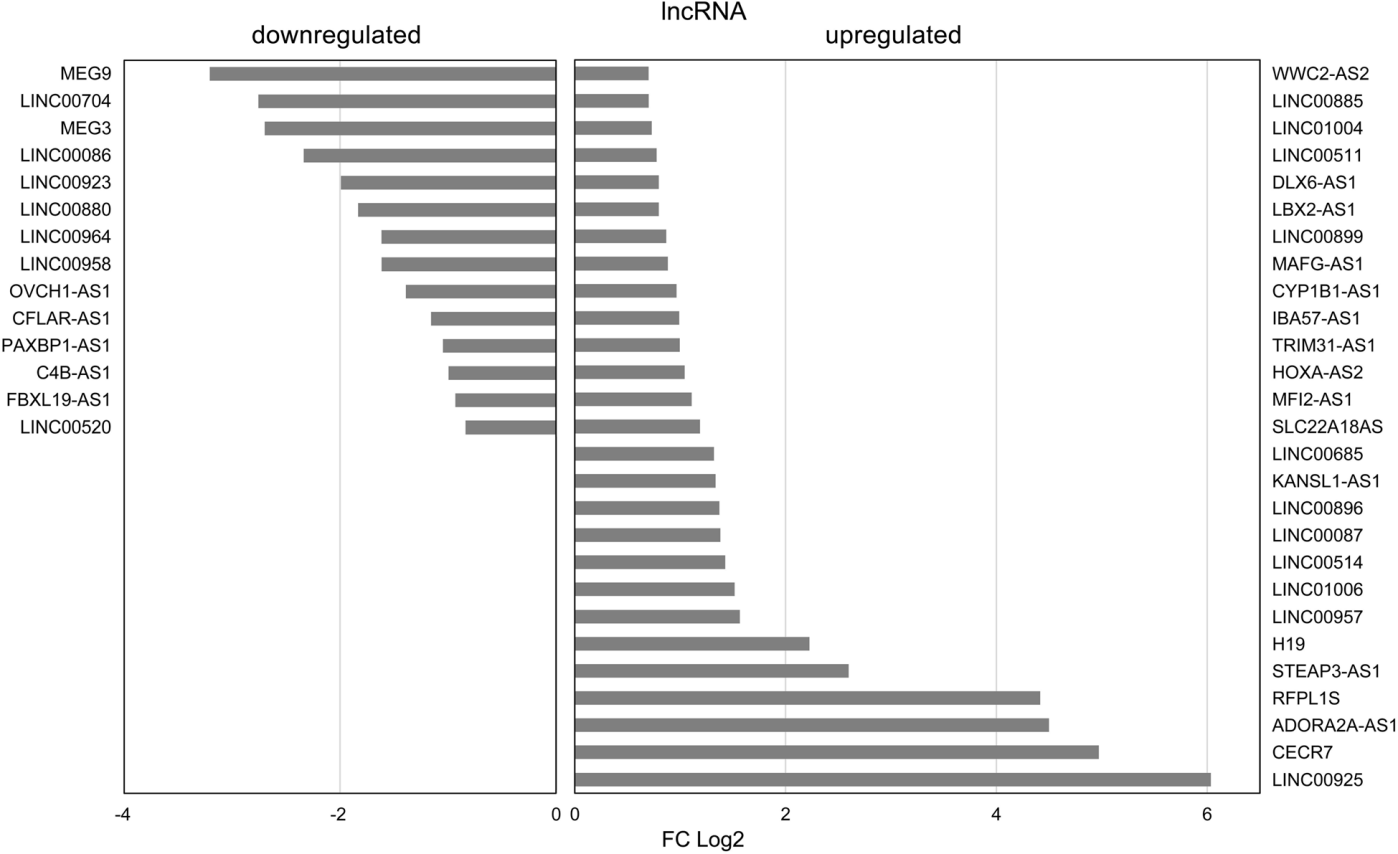
Differentially expressed lncRNAs in HPV16 E6/E7 expressing keratinocytes compared to control vector cells. Average Log2 transformed FC values of up - and downregulated lncRNAs are shown

41 differentially expressed lncRNAs were detected in E6/E7 expressing HFKs compared to control vector cells (E6/E7 vs. LXSN). Of these, 14 lncRNAs were downregulated (e.g. MEG3, MEG9, LINC00520, LINC00923, FBXL19-AS1) and 27 lncRNAs (e.g. H19, CECR7, LINC00087, LINC00925, DLX6-AS1, HOXA-AS2) were upregulated (Fig. 3).

**Fig. 4:**
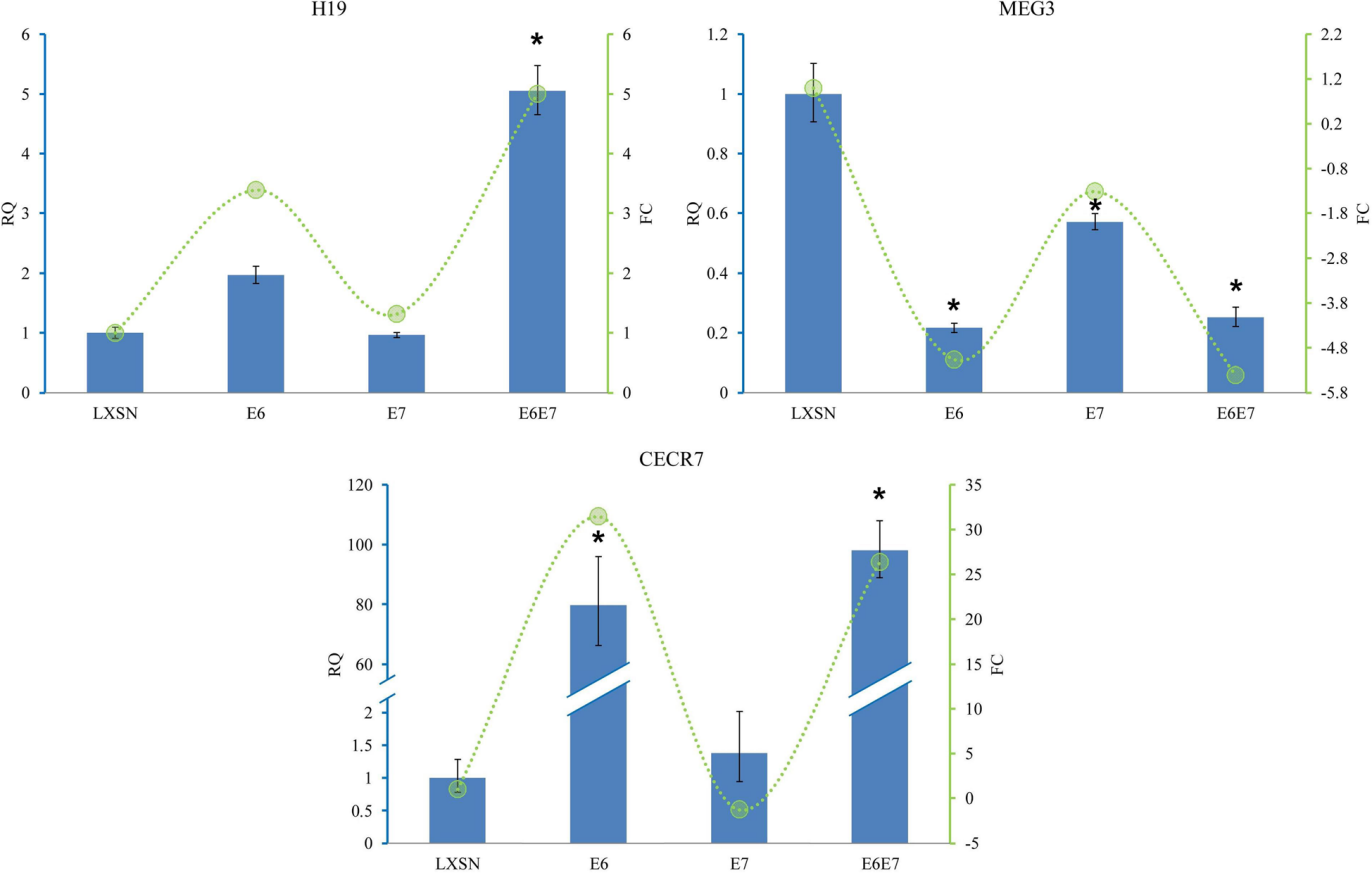
Validation of selected lncRNAs identified by large RNA seqencing. The integrated results of sequencing and TaqMan analyses of lncRNAs are shown. Green lines represent FC values obtained in large RNA sequencing. Altered expression of selected lncRNAs was confirmed using TaqMan real-time assays in HPV16 E6, HPV16 E7, HPV16 E6/E7 and LXSN control cells in three independent experiments. In data analysis, comparative C_t_ method was used to obtain the Relative Quantification (RQ) values with standard deviation and confidence intervals (95%). The RQ value of LXSN control was set to 1. Representative graphs are shown. *p < 0.01

TaqMan assays of selected lncRNAs also confirmed the results of sequencing. Significant downregulation of MEG3 and upregulation of CECR7 and H19 are represented on Fig. 4.

We applied the same criteria that were used in the case of miRNAs when we investigated the influence of individual oncoproteins on lncRNA expression changes. Like in the case of miRNAs, majority of the modifications in lncRNA expressions was either the dominant effect of E6 oncoprotein alone or the outcome of the presence of E6 and E7 together. The importance of E7 individually was observed only in two cases, while expressions of 21 lncRNAs were altered predominantly by E6 and expression changes of 18 lncRNAs were driven by the two oncoproteins together (Additional file 4).

### Coordinated action of HPV16 E6/E7 oncoproteins on ceRNA members: construction of regulatory network, GO and KEGG analyses

Predicted pairwise lncRNA-miRNA interactions were identified between the 85 differentially expressed miRNAs and the 41 differentially expressed lncRNAs using StarBase v2.0 and DianaTools-LncBase v.2 experiment - supported databases. As a result, 43 of 85 specific miRNAs and 15 of 41 specific lncRNAs were identified to interact in this experimental system. A total of 77 interactions between 15 lncRNAs and 43 miRNAs were identified based on combined results of database search. MEG3 lncRNA was found the most potent interaction partner with 29 miRNAs, followed by H19 lncRNA with 17 different miRNA interactions. Among miRNAs, miR-20b-5p, miR-143-3p, miR − 497-5p and miR-34a-5p, miR-195-5p were the most common molecules with interactions with 5 and 4 different lncRNAs, respectively (Table 2).

**Table 2:** lncRNA – miRNA interactions with potential functional significance identified in HPV16 E6/E7 expressing HFKs

lncRNAs	miRNAs	
	upregulated	downregulated
**upregulated**		
CECR7	hsa-miR-139-5p, hsa-miR-335-5p	hsa-miR-34a-5p
H19	hsa-miR-10a-5p, hsa-miR-20b-5p, hsa-miR-27b-5p, hsa-miR-99a-3p, hsa-miR-106a-5p, hsa-miR-143-3p, hsa-miR-193a-3p, hsa-miR-196b-5p, hsa-miR-342-3p, hsa-miR-615-5p	hsa-miR-100-3p, hsa-miR-100-5p, hsa-miR-125b-5p, hsa-miR-127-5p, hsa-miR-138-5p, hsa-miR-370-3p, hsa-miR-1271-5p
LINC00087	hsa-miR-20b-5p, hsa-miR-106a-5p	
LINC00511	hsa-miR-195-5p, hsa-miR-363-3p, hsa-miR-497-5p	hsa-miR-34c-3p
LINC00925	hsa-miR-139-5p	
LINC00957	hsa-miR-20b-5p	
DLX6-AS1	hsa-miR-132-3p, hsa-miR-195-5p, hsa-miR-20b-5p, hsa-miR-143-3p, hsa-miR-497-5p	hsa-miR-432-5p
MAFG-AS1	hsa-miR-143-3p	
MFI2-AS1	hsa-miR-497-5p	
RFPL1S	hsa-miR-143-3p	hsa-miR-136-3p, hsa-miR-34a-5p, hsa-miR-432-5p
**downregulated**		
LINC00086	hsa-miR-195-5p, hsa-miR-224-5p, hsa-miR-497-5p	
LINC00520		hsa-miR-34a-5p
LINC00923	hsa-miR-375	hsa-miR-34a-5p, hsa-miR-100-5p
MEG3	hsa-miR-20b-5p, hsa-miR-27b-5p, hsa-miR-99a-3p, hsa-miR-106a-5p, hsa-miR-132-3p, hsa-miR-143-3p, hsa-miR-181b-5p, hsa-miR-181c-5p, hsa-miR-195-5p, hsa-miR-224-5p, hsa-miR-342-3p, hsa-miR-362-5p, hsa-miR-363-3p, hsa-miR-497-5p, hsa-miR-542-3p, hsa-miR-3605-5p	hsa-miR-34a-3p, hsa-miR-100-5p, hsa-miR-127-3p, hsa-miR-136-3p, hsa-miR-221-5p, hsa-miR-377-5p, hsa-miR-409-5p, hsa-miR-432-5p, hsa-miR-485-3p, hsa-miR-501-3p, hsa-miR-539-3p, hsa-miR-543, hsa-miR-1271-5p
FBXL19-AS1	hsa-miR-342-3p	

**Fig. 5:**
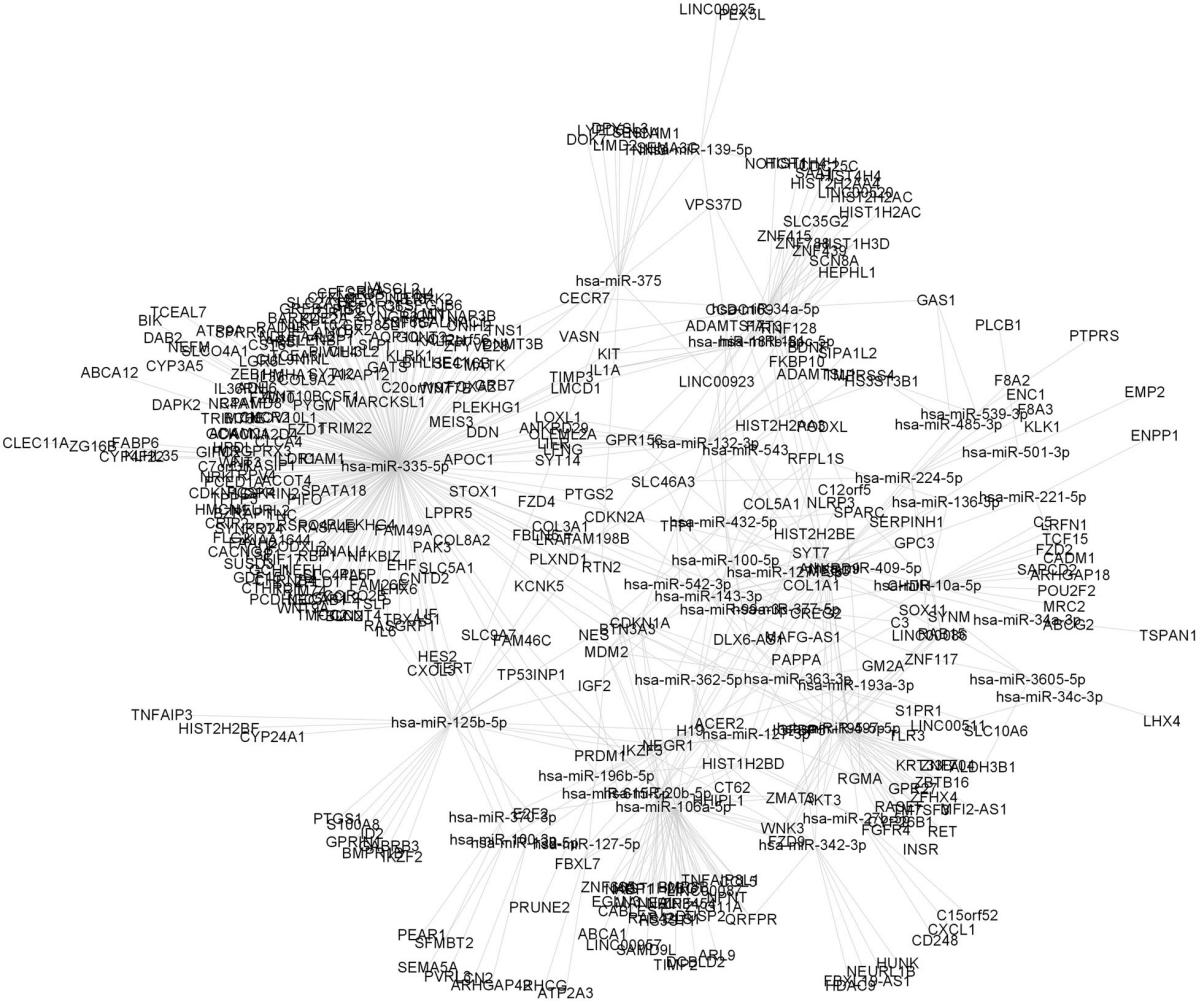
Construction of ceRNA network of differentially expressed lncRNAs-miRNAs-mRNAs in HPV16 E6/E7 expressing HFKs. Interactions of 15 lncRNAs – 43 miRNAs – 358 mRNAs are represented. The regulatory network was visualized with Cytoscape v3.4.0

To construct the whole lncRNA-miRNA-mRNA regulatory network, we searched mRNAs targeted by miRNAs using the miRTarBase. We mapped miRNA - mRNA pairs with experimental evidence using the advanced search function of miRTarBase. We investigated the 43 miRNAs that are in interactions with lncRNAs and mRNAs which showed differential expressions by HPV16 E6/E7. A total of 358 mRNAs targeted by 43 miRNAs were identified with expressions changed by E6/E7 oncoproteins (at least ±1.5 fold change in E6/E7 HFKs similarly to miRNAs and lncRNAs) and a total of 522 miRNA-mRNA pairs were observed (Table 3). After combination of lncRNA-miRNA and miRNA-mRNA interactions, we constructed a ceRNA network with members of 15 lncRNAs – 43 miRNAs – 358 mRNAs with significantly altered expressions driven by HPV16 oncoproteins. The regulatory network was visualized with Cytoscape v3.4.0. (Fig. 5).

**Table 3:** miRNA-mRNA target pairs with potential functional significance identified in HPV16 E6/E7 expressing HFKs

miRNAs	mRNA targets	
	upregulated	downregulated
**upregulated**		
hsa-miR-106a-5p	ARL9, BMP8B, CABLES1, CCL5, DUSP2, EGLN3, FZD9, MANEAL, NPNT, NRIP3, QRFPR, RAB42, RTN2, TIMP2, TNFAIP8L1, WNK3, ZNF454, ZYG11A	ACER2, CDKN1A, DCBLD2, FBXL7, HIST1H2BD, HIST1H2BG, HS3ST1, MDM2, NABP1, PLS1, TP53INP1, ZMAT3, ZNF665
hsa-miR-10a-5p	ABCG2, C5, CHDH, FZD2, LRFN1, MRC2, RAB15, SAPCD2, SPARC, TCF15	ARHGAP18, CADM1, POU2F2, TFPI
hsa-miR-132-3p	CCDC169, FKBP10, HS3ST3B1, LFNG, LOXL1, OLFML2A, SYT14	ADAMTSL1, ANKRD29, BDNF, CDKN1A, FAT3, LIFR, PTGS2, RNF128, TFPI
hsa-miR-139-5p	ADAMTS17	NOTCH1, PEX5L
hsa-miR-143-3p	ANKRD9, COL1A1, COL3A1, COL5A1, CREG2, IGFBP5, IKZF3, LRAT, SYT7	MDM2, PAPPA, PTGS2, TFPI
hsa-miR-181b-5p	LMCD1, SCN8A, SLC35G2, TIMP3, ZNF415	HEPHL1, HIST1H3D, IL1A, ZNF439, ZNF788
hsa-miR-181c-5p	KIT, SCN8A, SLC35G2, ZNF415	HEPHL1, HIST1H3D, IL1A, ZNF439, ZNF788
hsa-miR-193a-3p	IGFBP5, RGMA	C12orf5, SLC10A6, ZMAT3
hsa-miR-195-5p	AKT3, ALDH3B1, CYP26B1, FGFR4, FZD9, GPR27, INSR, RAB15, RASEF, RET,, WNK3, ZBTB16, ZNF704	BTN3A3, CDKN1A, HIST2H2BE, KRT33B, NEGR1, TM7SF3, ZFHX4, ZMAT3
hsa-miR-196b-5p	PRUNE2	ACER2, HIST1H2BD, SLC9A7
hsa-miR-20b-5p	BMP8B, CABLES1, CCL5, DUSP2, E2F2, EGLN3, FAM46C, FZD9, MANEAL, NPNT, NRIP3, QRFPR, RAB42, RTN2, TNFAIP8L1, WNK3, ZNF454, ZYG11A	ABCA1, ACER2, BTN3A3, CDKN1A, FBXL7, HIST1H2BD, HIST1H2BG, HS3ST1, MDM2, NABP1, PLS1, SAMD9L, TP53INP1, ZMAT3, ZNF665
hsa-miR-224-5p	F8A2, F8A3, KLK1, RAB15	ENC1, SLC46A3
hsa-miR-27b-5p	CD248	C15orf52, CXCL1, HIST1H2BD
hsa-miR-335-5p	ACOT4, AKAP12, APOC1, AQP10, ARSI, ASCL2, BZRAP1, C12orf56, C7orf31, CACNA2D4, CACNG4, CDKN1C, CELSR3, CHRNB4, CLEC11A, CLMN, CNIH2, CNTD2, COL3A1, COL8A2, COL9A2, CPAMD8, CRIP2, CTHRC1, CTSF, CTXN1, DAPK2, DDN, DNALI1, DNMT3B, FAM46C, FBLN2, FBLN5, FZD1, FZD10, GATS, GCK, GIPC2, GJB6, GPR156, GPRIN2, GRB7, HMHA1, HPDL, ICAM1, KCNK5, KIAA1644, KIF17, KIT, KLHL35, LFNG, LGR6, LHX6, LMCD1, LOXL1, LRAT, LSP1, MARCKSL1, MATK, MEIS3, MOV10L1, NDUFA4L2, NECAB1, NEFH, NEFM, NINL, NR4A1, NRK, OLFML2A, PCED1A, PCSK4, PDE2A, PIWIL4, PLEKHG4, PLLP, PLXND1, PODXL2, PPP1R36, PYGM, RAD9B, RASA4B, RASIP1, RBP1, RGCC, RSPO4, RTN2, SELENBP1, SLCO4A1, STOX1, SUSD3, SYNGR3, SYNPO2, SYT12, SYT14, TBXAS1, TCEA3, TERT, TIMP3, TMCC2, TNC, TNS1, TPPP3, TRIM73, TRIM74, TRPV4, TSLP, VASN, WNT10B, WNT3, WNT7B, WNT9A, YBX2, ZFYVE28, ZG16B, ZNF157	ABCA12, ADAM21, ADH6, ANKRD29, ANO1, ATP9A, BARX2, BCHE, BHLHE41, BIK, BTN3A3, C20orf197, CDKN1A, CEP85L, CHI3L2, CLCA4, CNTNAP3B, CORO2B, CUL9, CXCL2, CXCL3, CXCR2, CYP3A5, CYP4F22, CSF1, CST6, DAB2, EHF, FAAH2, FABP6, FAM198B, FAM26E, FAM49A, FCRLA, FLG2, FOXA2, GCH1, GCNT2, GCNT4, GDF15, GREB1, GRIP2, HES2, HMCN1, IL1A, IL33, IL36RN, IL6, ILDR1, IVL, KALRN, KLRK1, KRT4, LIF, LIFR, LPPR5, LRRK2, MDM2, MRGPRX3, NEURL2, NFKBIZ, NLRP10, PAK3, PCDHGC5, PIFO, PLEKHG1, PLIN4, PTGS2, RASGRP1, SEC16B, SERPINB10, SLC2A13, SLC44A5, SLC46A3, SLC5A1, SLC9A7, SLPI, SPATA18, SPRR3, ST6GALNAC1, TCEAL7, TFPI, TP53INP1, TRIM22, ZEB1, ZPLD1
hsa-miR-342-3p	HUNK, IGFBP5, IKZF3, EURL1B, QRFPR	HDAC9
hsa-miR-3605-5p	ALDH3B1	LHX4, ZNF117
hsa-miR-362-5p	HHIPL1, KCNK5	CT62
hsa-miR-363-3p	SOX11	CDKN1A, GM2A, MDM2, S1PR1, TLR3
hsa-miR-375	DNMT3B, DOK7, DPYSL3, FZD4, LIMD2, NCAM1, RTN2, TNNI3, TNS1, VASN	LYPD5, SEMA3C, SESN1, VPS37D
hsa-miR-497-5p	AKT3, ALDH3B1, CYP26B1, FGFR4, FZD9, GPR27, RAB15, RASEF, WNK3, ZBTB16, ZNF704	BTN3A3, HIST2H2BE, KRT33B, NEGR1, TM7SF3, ZFHX4, ZMAT3
hsa-miR-542-3p	FBLN5	MDM2, TFPI
hsa-miR-615-5p	IGF2	–
hsa-miR-99a-3p	–	SLC46A3
**downregulated**		
hsa-miR-100-3p	SEMA5A	CXCL3, PVRL3
hsa-miR-100-5p	IGF2	HIST2H2AA3, NLRP3
hsa-miR-125b-5p	BMPR1B, CDKN2A, CYP24A1, E2F2, FZD4, GPRIN1, IGF2, IKZF3, NES, PLXND1, PTGS1, TBXAS1	GABRB3, HIST2H2BF, ID2, IKZF2, IL6, LIF, NEGR1, PRDM1, RASGRP1, S100A8, TNFAIP3, TP53INP1
hsa-miR-1271-5p	GPC3, GPR156, PODXL, SERPINH1, SYNM	ACER2
hsa-miR-127-3p	RGMA	PRDM1
hsa-miR-127-5p	ATP2A3	MDM2, RHCG
hsa-miR-136-3p	SOX11	–
hsa-miR-138-5p	TERT	ARHGAP42, LCN2
hsa-miR-221-5p	ENPP1, FZD2	–
hsa-miR-34a-3p	–	TSPAN1
hsa-miR-34a-5p	CDC25C, CDKN2A, GAS1, GPR156, KIT, PODXL, SLC35G2, VASN	C12orf5, HIST1H2AC, HIST1H3D, HIST1H4H, HIST2H2AA3, HIST2H2AA4, HIST2H2AC, HIST2H2BE, HIST4H4, NOTCH1, SAA1, VPS37D
hsa-miR-34c-3p	SYNM	–
hsa-miR-370-3p	SFMBT2	HES2, PEAR1
hsa-miR-377-5p	CHDH	C12orf5, C3, FAM198B, GM2A, HIST1H2BD, TFPI, ZNF117
hsa-miR-432-5p	ADAMTS17, NES, SPARC	MDM2, TFPI
hsa-miR-485-3p	GAS1, HS3ST3B1	–
hsa-miR-501-3p	EMP2	PLCB1
hsa-miR-539-3p	GAS1, HS3ST3B1, PTPRS	–
hsa-miR-543	ADAMTS17, COL5A1, PLXND1, TMPRSS4	SIPA1L2

In order to understand the biological processes affected by ceRNA regulatory network, the mRNAs were submitted to GO and KEGG analyses using DAVID v6.8 database. Functional enrichment analysis revealed 95 of 358 genes as cancer – associated genes, such as E2F2, MDM2, CDC25C, CDKN1A, CDKN1C, CDKN2A, GAS1, IGF2, NOTCH1, TERT etc.. According to KEGG analysis the genes in the regulatory network are involved in pathways such as pathways in cancer, signaling pathways regulating pluripotency of stem cells, proteoglycans in cancer, microRNAs in cancer, TNF signaling pathway, Hippo signaling pathway, viral carcinogenesis, Wnt signaling pathway. In the GO analysis (GO, level BP3), key biological processes which may play important roles in HPV-induced carcinogenesis were significantly enriched such as signal transduction, regulation of cell communication, regulation of cell cycle, cell cycle arrest, cell death, regulation of cell death, cell migration, regulation of cell proliferation, epithelial cell proliferation, etc.. The lists of genes significantly enriched GO and KEGG pathways are reported as additional file (Additional file 5).

**Fig. 6:**
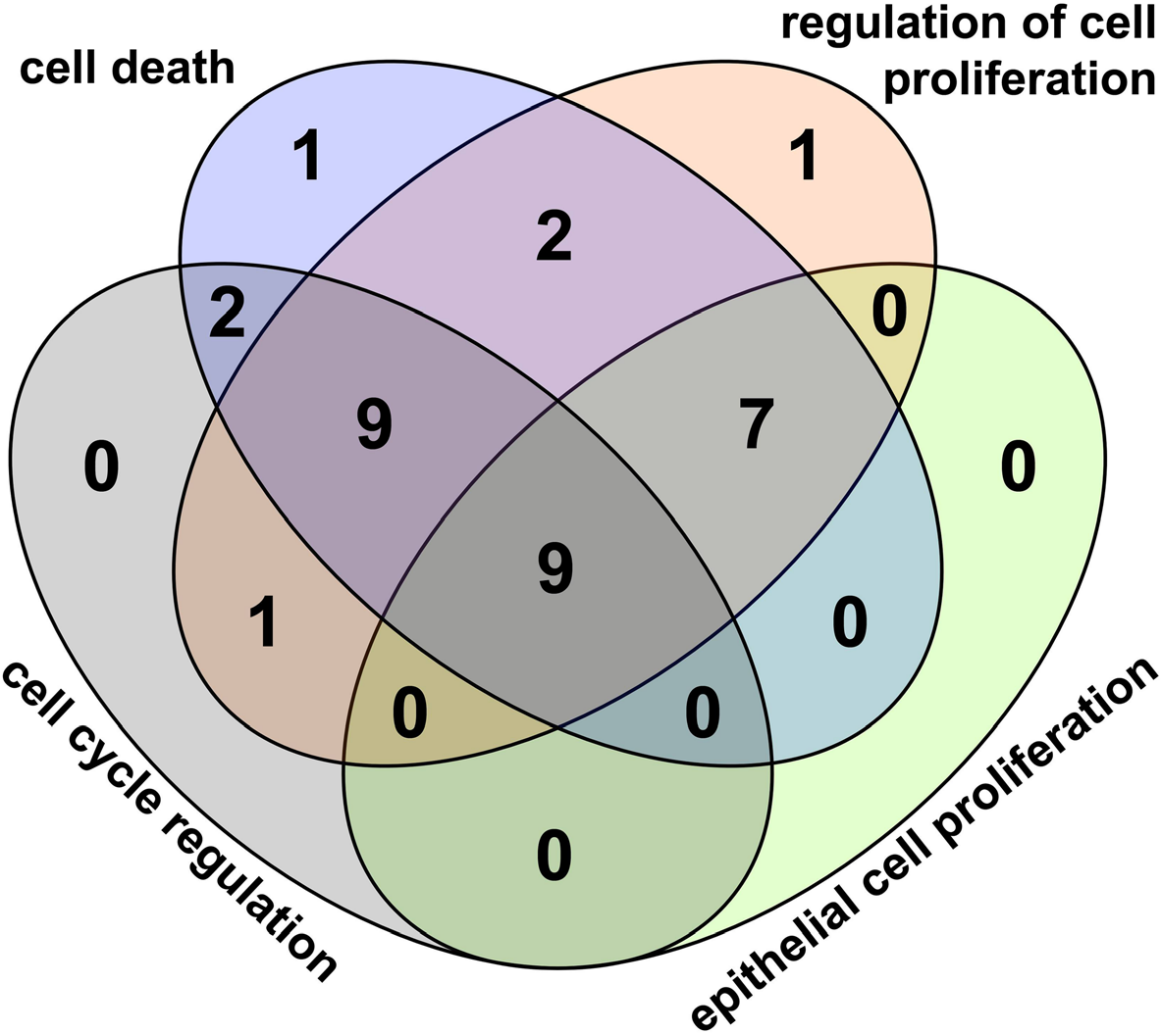
Venn diagram of dysregulated miRNAs involved in particular biological processes with significance in cancer development

**Fig. 7:**
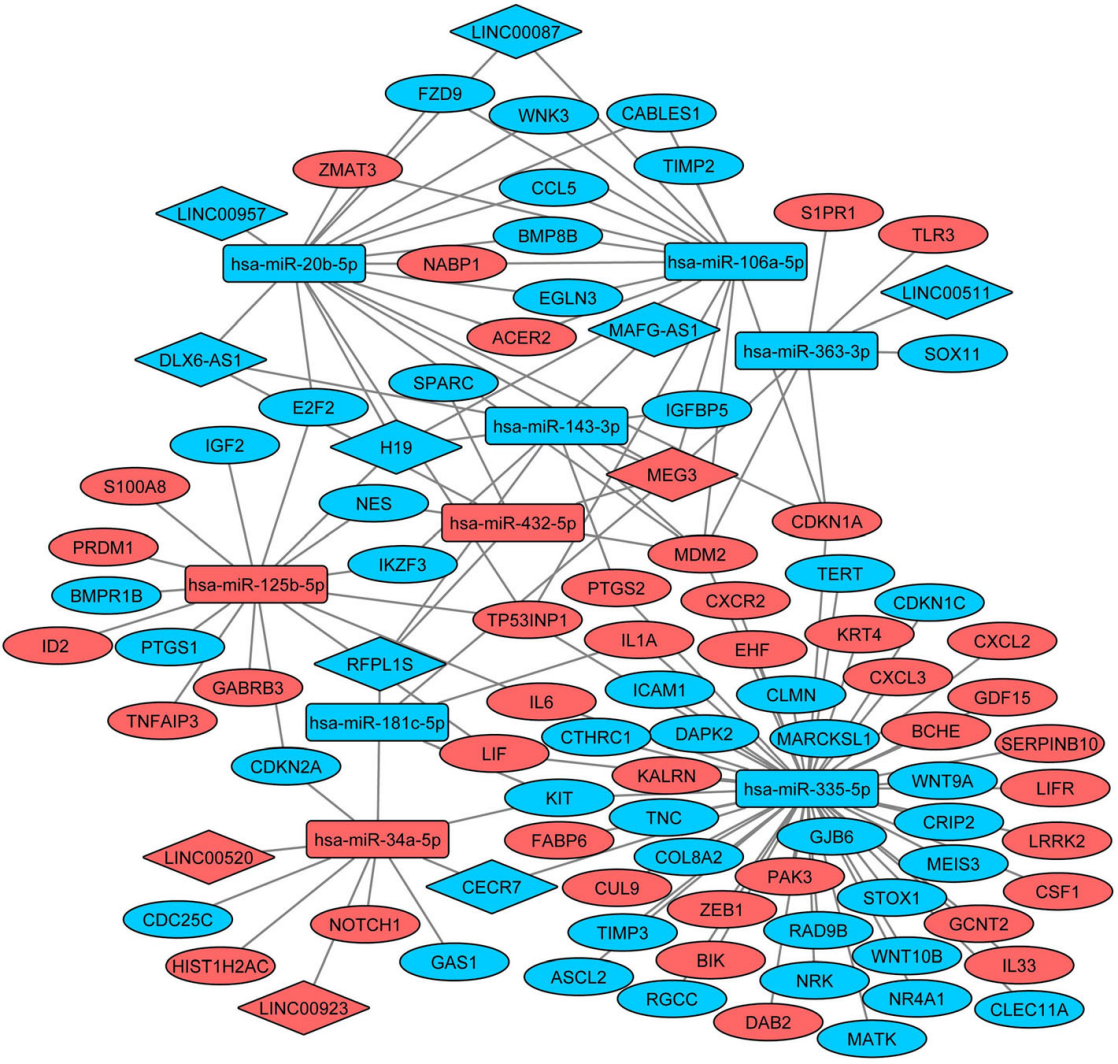
The integrated ceRNA network of cancer related biological processes in HPV16 E6/E7 expressing cells. The network consists of 11 lncRNAs and 82 mRNAs targeted by nine common miRNAs in the regulatory modules. Diamonds represent lncRNAs, rectangles represent miRNAs and ovals represent mRNAs. RNAs with red were downregulated and RNAs with blue were upregulated. The regulatory network was visualized with Cytoscape v3.4.0

We examined the effect of HPV16 E6/7 on the expression of ceRNA members involved in specific biological processes important in cancer development to see if there are „hot points” in the biological activity of oncoproteins. Nine common miRNAs targeting mRNAs which are components of key canonical pathways were identified. miR − 20b-5p, miR-34a-5p, miR-106a-5p, miR-125b-5p, miR-143-3p, miR-181c-5p, miR-335-5p, miR-363-3p and miR-432-5p were observed as key regulatory elements in regulation of cell cycle and cell death, regulation of cell proliferation, regulation of epithelial cell proliferation, in such biological processes which have an impact in cancer development (Table 4, Fig. 6). The integrated ceRNA network of above mentioned biological processes consists of 11 lncRNAs and 82 mRNAs targeted by common miRNAs in the regulatory modules (Fig. 7). These results support the existence of regulatory „hot points” in key cancer related pathways driven by the coordinatied action of HPV16 E6 and E7 oncoproteins on ceRNA members.

**Table 4:** Common miRNAs with functional importance in four cancer related regulatory pathways

Biological process	Number of common miRNAs	Common miRNAs
cell death + cell cycle regulation + epithelial cell proliferation + regulation of cell proliferation	9	hsa-miR-432-5p, hsa-miR-20b-5p, hsa-miR-106a-5p, hsa-miR-34a-5p, hsa-miR-181c-5p, hsa-miR-335-5p, hsa-miR-143-3p, hsa-miR-363-3p, hsa-miR-125b-5p
cell death + cell cycle regulation + regulation of cell proliferation	9	hsa-miR-181b-5p, hsa-miR-138-5p, hsa-miR-497-5p, hsa-miR-127-5p, hsa-miR-100-5p, hsa-miR-195-5p, hsa-miR-501-3p, hsa-miR-542-3p, hsa-miR-132-3p
cell death + epithelial cell proliferation + regulation of cell proliferation	7	hsa-miR-193a-3p, hsa-miR-139-5p, hsa-miR-342-3p, hsa-miR-1271-5p, hsa-miR-100-3p, hsa-miR-136-3p, hsa-miR-10a-5p
cell death + cell cycle regulation	2	hsa-miR-485-3p, hsa-miR-539-3p
cell cycle regulation + regulation of cell proliferation	1	hsa-miR-615-5p
cell death + regulation of cell proliferation	2	hsa-miR-196b-5p, hsa-miR-27b-5p
cell death	1	hsa-miR-3605-5p
regulation of cell proliferation	1	hsa-miR-127-3p

## Discussion

E6 and E7 oncoproteins of high-risk HPVs cause large-scale gene expression changes in the HPV infected epithelial cells. They interact with numerous signaling molecules, thereby they are able to reprogram multiple cellular regulatory pathways involved in cell cycle, cell death, cell differentiation and migration playing a crucial role in HPV-induced abnormal cell proliferation and malignant transformation [5, 58, 60]. In addition to protein coding genes, E6 and E7 can also alter the expression of non-coding RNAs, including lncRNAs and miRNAs [29, 39].

Host cell’s non-coding RNAs, especially miRNAs and lncRNAs function as key regulators of cellular biological processes both under physiological and pathological conditions, including tumour initiation and progression by multiple types of cancers including HPV-associated malignancies. However, the majority of the studies have focused on high-risk HPV associated expressional changes and/or interactions of either miRNAs or lncRNAs. Several studies indicated that miRNAs and lncRNAs may interact with and regulate cellular processes involved in cancer development through modulation of the expression of their downstream mRNA target [47, 48, 61]. Nevertheless, non-coding RNA molecules, both miRNAs and lncRNAs, have the potential to interact many other RNAs and modulate numerous mRNA targets and vice versa, a single mRNA may be targeted by hundreds of miRNAs. Therefore, the aberrant expression of these RNA molecules will influence multiple signaling pathways in HPV infected cells. Thus, analyzing the global context of coding and non-coding RNAs provides valuable information on the complexity of the structure and function of endogenous RNA network regarding high-risk HPV infection. Therefore our goal was to investigate the global expression changes of miRNAs, lncRNAs and mRNAs driven by HPV16 E6/E7 oncoproteins. We described the network of abnormally expressed non-coding RNAs in stably transduced undifferentiated human primary keratinocytes using small RNA (≤ 200 nt) and large RNA (≥ 200 nt) deep sequencing. To understand the potential impact of expression alterations of miRNAs and lncRNAs modulated by E6/E7 oncoproteins on cellular mRNA abundance and functions, we have paired the expression data of the various RNA populations. Furthermore, additional GO and KEGG analyses were performed to understand the consequences of mRNA expression alterations on biological processes. To our best knowledge, to date this is the first study which describes such an integrated expression data of non-coding RNAs and mRNAs and construct a ceRNA network of miRNAs, lncRNAs and mRNAs in association with high-risk HPV infection. It is important to highlight, that all of our functional analyses were performed on the basis of E6/E7 expressing HFK cells, because this scenario represents best the in vivo HPV16 infection in epithelial cells. Beyond that, we examined the individual effect of E6 and E7 oncogenes on non-coding RNA expression, too. It is well documented, that in high-risk HPV-types, including HPV16, E6 and E7 expression is controlled by alternative splicing resulting in three different E6 isoforms (E6*I, E6*II and E6^E7) besides the unspliced full-length E6. The expression of different HPV16 isoforms may vary among cells and tissues, nevertheless E6*I is reported to be the most abundant isoform in cervical tumors and cells [62]. Two different E6 products were detected in HPV16 E6 specific RT-PCR performed in this study which matched the full-length E6 and an E6 splice isoform. Only the full length E6 transcript is responsible for coding the transforming E6 oncoprotein while different E6 isoforms may have an antagonistic effect in tumorigenesis. However, both the regulation of these isoforms and their effect on non-coding RNA expression is poorly understood, there are limited number of studies reporting relationship between miRNAs and E6 isoforms [63]. Although, we were not able to differentiate the effect of full-length E6 and E6 splice isoform on the expression of different RNA populations in our study, our observations likely show the cumulative effect of different E6 isoforms and E7 oncoprotein on RNA expression in our experimental system which may be significant in tumorigenesis. At the same time, further research needs to be performed to clarify the exact role of E6 isoforms on non-coding RNA expression in HPV16 host cells.

In our study, we revealed that 85 miRNAs and 41 lncRNAs were abnormally expressed in HPV E6/E7 expressing cells compared with controls. When we examined the impact of oncoproteins on RNA alteration profile individually, we found that the vast majority of RNA expression changes was driven primarly by E6 oncoprotein, which was not a surprise as E6 has a strong effect on host cell processes [64]. In total, the expression of 21 miRNA clusters were modulated by HPV16 E6/E7 oncoproteins, affecting 55 differentially expressed miRNAs. According to chromosome location, 38 miRNA members of miRNA clusters on chromosome X and chromosome 14 may have functional importance in HPV16 infected cells, seeing the clustered miRNAs often coordinately regulate certain biological processes. Harden and coworkers also described modulation of multiple miRNA clusters by HPV16 E6/E7 oncoproteins and they found several miRNAs with expression changes on chromosome X and chromosome 14 [29].

This study also revealed that several differentially expressed non-coding RNAs tend to have regulatory interaction with multiple RNA species also altered in the presence of HPV16 oncogenes. We constructed a ceRNA network with members of 15 lncRNAs – 43 miRNAs – 358 mRNAs with significantly altered expressions driven by HPV16 oncoproteins. Among non-coding RNAs, lncRNAs such as MEG3 and H19, and miRNAs such as miR − 20b-5p, miR-143-3p, miR-497-5p, miR-34a-5p and miR-195-5p had the highest number of relationships in the interaction network. In accordance with our results, downregulation of MEG3 and overexpression of H19 lncRNAs were found by several research groups in association with various neoplasms and the link of these lncRNAs with p53 and pRb pathways underlines their significance in E6 and E7 mediated transformation [19, 65, 66]. The relationships between lncRNAs and miRNAs, and miRNAs and mRNAs were predicted by using StarBase v2.0, DianaTools-LncBase v.2 and miRTarBase, which are experiment - supported databases corroborating that the RNA-RNA interactions identified in our experiment would occur not only in silico situation [53–55]. These supporting data justified the construction of a ceRNA network from the experimental data of this study.

The functional enrichment analyses identified numerous ceRNA member coding genes as cancer related genes and the majority of mRNAs are involved in GO biological processes and KEGG pathways associated with cancer initiation and progression. Furthermore, our comprehensive examination recognized nine miRNAs as key regulatory elements in regulation of cell cycle and cell death, regulation of cell proliferation and regulation of epithelial cell proliferation. Although, all of these miRNAs have been reported in previous studies associated with HPV infection, their functions in such a complex ceRNA network were so far unknown and they are worth to be further investigated as potential biomarkers in disease detection and progression.

## Conclusions

In this study, our main goal was to highlight the complexity of regulatory network of cellular non-coding RNAs in HPV16 infected cells. In the last few years, meta-analyses of sequencing data from different databases or analyzing the global context of coding and non-coding RNAs using integrative methods have become a new and widespread direction of current cancer research. Nevertheless, carrying further experiments focusing on real biological impact of certain RNA-RNA interactions is very useful and essential to explore the exact functions and regulations in depth of non-coding RNAs in cancer development.

We hypothesize, that the multiple molecular changes driven by E6 and E7 oncoproteins resulting in the malignant transformation of HPV16 infected host cells may occur, at least in part, due to the abnormal alteration in expression and function of non-coding RNA molecules through their intracellular competing network. Although several lncRNAs and miRNAs in this study have been previously reported regarding both to HPV-associated and non-HPV cancers, to our best knowlege, this is the first study which describes a ceRNA network of lncRNAs-miRNAs-mRNAs in HPV16 infected cells. Functional enrichment analyses carried out in this study assume important regulatory roles of certain miRNAs and lncRNAs in key biological processes involved in tumour development. We believe, that our comprehensive results will provide a basis for further detailed experiments. Based on these expressional data, we plan to perform further experiments in the future to investigate the relationships of certain non-coding RNAs which funtion is so far poorly understood. Furthermore, miRNAs and lncRNAs can serve as promising biomarkers for molecular diagnosis and progression of cervical cancers.

## Supplementary Information

**Additional file 1.** The lists of StarBase v2.0, DianaTools-LncBase v.2 and miRTarBase search results.

**Additional file 2.** Result of HPV16 E6 and E7 specific RT-PCR.

**Additional file 3.** The effects of the HPV16 E6 and E7 on the level of well-known transcriptional targets of high-risk HPV oncoproteins.

**Additional file 4.** Influence of individual HPV16 oncoproteins on miRNA and lncRNA expression changes.

**Additional file 5.** The lists of genes significantly enriched GO and KEGG pathways.

## Data Availability

The datasets used and/or analysed during the current study are available from the corresponding author on reasonable request.

## Abbreviations

HPV: Human papillomavirus
miRNA: microRNA
lncRNA: long non-coding RNA
ceRNA: competitive endogenous RNA
MRE: microRNA response element
HFK: Human foreskin keratinocyte
GO: Gene Ontology
KEEG: Kyoto Encyclopedia of Genes and Genomes
