# Supplementary figures and images for: Coordinated action of human papillomavirus type 16 E6 and E7 oncoproteins on competitive endogenous RNA (ceRNA) network members in primary human keratinocytes
Source: BMC Cancer. 2021 Jun 7;21:673. doi: 10.1186/s12885-021-08361-y (PMC8185923; doi:10.1186/s12885-021-08361-y)

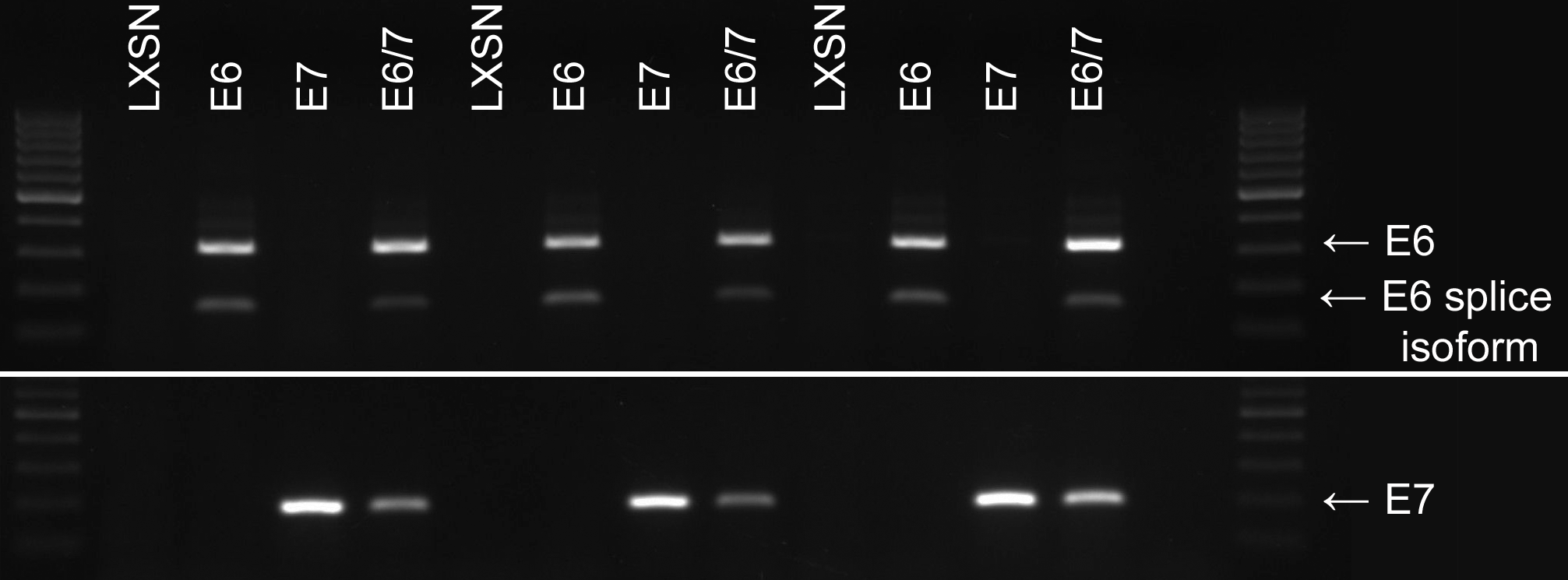

Supplement: Supplementary file 2 — Additional file 2. Result of HPV16 E6 and E7 specific RT-PCR. [file 12885_2021_8361_MOESM2_ESM.tif]
